# Live Bacterial Vectors—A Promising DNA Vaccine Delivery System

**DOI:** 10.3390/medsci6020027

**Published:** 2018-03-23

**Authors:** Valentina Yurina

**Affiliations:** Department of Pharmacy, Medical Faculty, Universitas Brawijaya, East Java 65145, Malang, Indonesia; v_yurina@ub.ac.id; Tel.: +62-341-569-117

**Keywords:** lactic acid bacteria, antigen expression, oral delivery, carrier

## Abstract

Vaccination is one of the most successful immunology applications that has considerably improved human health. The DNA vaccine is a new vaccine being developed since the early 1990s. Although the DNA vaccine is promising, no human DNA vaccine has been approved to date. The main problem facing DNA vaccine efficacy is the lack of a DNA vaccine delivery system. Several studies explored this limitation. One of the best DNA vaccine delivery systems uses a live bacterial vector as the carrier. The live bacterial vector induces a robust immune response due to its natural characteristics that are recognized by the immune system. Moreover, the route of administration used by the live bacterial vector is through the mucosal route that beneficially induces both mucosal and systemic immune responses. The mucosal route is not invasive, making the vaccine easy to administer, increasing the patient’s acceptance. Lactic acid bacterium is one of the most promising bacteria used as a live bacterial vector. However, some other attenuated pathogenic bacteria, such as *Salmonella* spp. and *Shigella* spp., have been used as DNA vaccine carriers. Numerous studies showed that live bacterial vectors are a promising candidate to deliver DNA vaccines.

## 1. Introduction

The DNA vaccine is a new vaccine with a bacterial plasmid as the antigen gene vector. Typically, the gene is expressed under host cell promoter regulation, and the expressed antigen is targeted to induce the host immune response [1]. DNA vaccine development is based on its advantages compared with conventional vaccines, such as the ability to induce a cellular immune response instead of only a humoral immune response [2]. The expressed antigen could be designed as an extracellular protein, which is detected by Major Histocompability Complex (MHC) class II or an intracellular protein that is recognized by MHC class I. DNA plasmid is also stable at room temperature, facilitating its production and distribution. DNA vaccine production does not directly use the pathogen organism, which is safer for the production system [1,2].

Some concerns about the DNA vaccine include potential genomic integration and auto-immune response [3]. However, extensive research found that the risk of integration is limited and significantly lower than the natural mutation rate [3,4,5]. Clinical trials also showed mild side effects after DNA vaccination [6,7,8].

Although promising, the DNA vaccine needs a superior delivering system to activate the potent immune system [9]. The DNA vaccine requires a large dose to effectively induce the immune system response [10]. At least 1–100 μg of DNA vaccine is needed to induce immune response [11]. Although some naked DNAs demonstrated efficacy [12,13,14], several delivery strategies have been studied to increase DNA vaccine efficiency. Chemical delivery systems, such as using micro particles [11,15] and nanoparticles [16,17], have successfully increased DNA vaccine delivery. Another potential delivery system to improve DNA vaccine delivery is the use of a live bacterial vector as the carrier.

Since being developed in the late 1970s, the idea of using live bacteria as the DNA carrier has been growing rapidly. The technique is also known as bactofection, in which the live bacteria are directly used to transfer the DNA to the target cells, tissues, or organs [18]. Bactofection has been widely used in the field of drug development research [19], such as cancer treatment [20], infections [21], inflammation diseases and other metabolic diseases [22,23]. As a DNA vaccine carrier, both native [24] or recombinant bacteria can be used. The live bacterial vector does not only deliver DNA inside the host cell but also induces a potent immune response due to its immunogenic features [25,26]. In this review, an overview of live bacterial vectors as DNA vaccine carriers and future prospects in this field is provided.

## 2. DNA Vaccine Components

**Figure 1:**
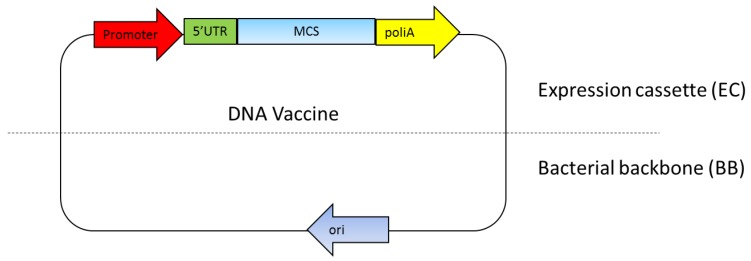
DNA vaccine components. The essential components in the DNA vaccines consist of a eukaryotic promoter, a multiple cloning site (**MCS**), a polyadenylation site (**polyA**), a selection marker and a bacterial origin of replication (**ori**). 5’UTR: 5′ untranslated region.

The DNA vaccine essentially includes two main parts: a mammalian expression cassette and bacterial backbone [27]. The mammalian expression cassette consists of a eukaryotic promoter for gene expression, 5′ untranslated region (5′UTR) including an intron and polyadenilation sequence (polyA) (Figure 1). The bacterial backbone consists of bacterial origin of replication (Ori) and an antibiotic resistance gene or other selection markers (Figure 1). Ideally, the multiple cloning sites (MCS) used to insert the target gene are located between the mammalian expression cassette and the bacterial backbone [27,28,29]. The mammalian expression cassette should be optimized so that the antigen gene is highly expressed to effectively generate the immune response [29]. The bacterial backbone should also be optimized so that a high yield of DNA plasmid can be produced with the fermentation production process [27,30]. The newest DNA vaccine version combines a component in the eukaryotic expression cassette while minimizing the bacterial backbone components, since it reduces antigen expression level [27,31].

The *Escherichia coli* Ori ColE1 found, for instance, as OriV of the pUC vector, is still a prominent choice of bacterial origin of replication because of its high copy number of up to 500–700 copies per bacterial cell [32]. This OriV was used in the early generation DNA vaccines, such as in pVAK1 to the latest generation of DNA vaccines, such as NTC8385 [33]. This suggests that this OriV is still considered as an ideal *ori* for DNA vaccines [29,30].

Selectable markers are required for the maintenance of the plasmid inside the cells. Only the cells that contain plasmids with the appropriate selectable marker can survive under the selective conditions. Most DNA plasmids are based on antibiotic resistance genes as the selectable markers. However, use of antibiotic resistance genes has resulted in health concerns being expressed, such as the spreading of the resistance genes and the effect on the microbiota in the host system [34]. Thus, the European Medicines Agency (MEA, London, UK) recommends non-antibiotic resistance genes as the selection marker. The antibiotic resistance gene is replaced with other marker selections based on an auxotrophic strain, toxin-antitoxin systems, operator-repressor titration, RNA markers, or the minicircles [31,35,36,37].

The promoter recognized by the mammalian expression system plays significant roles in antigen gene expression. Cytomegalovirus (CMV), Simian virus 40 (SV40), and murine leukemia virus promoters are among the most prominent promoters used in DNA vaccines [38,39]. Although CMV promoter activity decreases under some conditions, such as when used in conjunction with cytokine treatment [29], the combination of CMV promoter with Intron A showed that the CMV promoter effectively activates gene expression. Some studies also combined a CMV promoter with other components, such as a modified chicken β-actin [39] or woodchuck hepatitis post-transcriptional regulatory element (WPRE), to create a hybrid promoter [40]. Other non-viral promoters have been demonstrated to have comparable efficacy to the CMV promoter, such as collagen [41] and keratinocytes [42] promoters. Another study indicated that the MHC class II promoter potently generated a transgene product that can be used in the DNA vaccine [43].

The polyadenylation sequence has a significant effect on transgene expression. The common polyadenylation sequences used in DNA vaccine construction are SV40, rabbit β-globin, and bovine growth hormone polyadenylation sequence [29,44]. 5′UTR is located upstream transgene and regulates transgene translation. Optimization of the regulatory element by inserting a sequence from the R region of the long terminal repeat from human T-cell leukemia virus type 1 (HTLV-1) to CMV enhancer/promoter markedly increased DNA vaccine immunogenicity in both mice and non-human primates [45].

## 3. Live Bacterial Vector as the DNA Vaccine Carrier

As a DNA vaccine delivery system, the live bacterial vector has several benefits. The bacteria typically used for a delivery system are recombinant bacteria that have been genetically modified so most of their pathogenicity components have been deleted to attenuate the bacteria and create a non-virulent organism, ensuring the safety of the host [26]. By using bacteria as the carrier, the vaccination can be delivered through mucosal routes, including intranasal, oral, or intravaginal routes. The mucosal route is favorable because it is non-invasive and more acceptable. Administration through oral routes also does not require special skills and is easier to manage. Vaccination through mucosal routes induces both mucosal and systemic immune system responses [46,47]. Bacteria protect the DNA vaccine from harsh environments and enzymatic reactions in the gut [48]. The intranasal route has also been thoroughly developed since it can hinder enzymatic reactions and withstand the high acidity conditions in the gut. Other studies showed that vaccines administered through the intranasal route induce the same or better immune response compared with oral route vaccinations [47,49].

The mucosal surface is the first location where the host and its environment contact; therefore, it has a prominent defense mechanism against pathogens. Mucosal route vaccine delivery systems are based on mucosa-associated lymphoid tissue (MALT), which is found on various mucosal surface areas. MALT is a lymphoid tissue in the nasopharynx, pharynx, salivary gland, and upper respiratory tract, which are known as nasal-associated lymphoid tissues (NALT). MALT is also found in the broncho epithelium and lower respiratory tract (BALT), gastrointestinal tract (GALT) and genital tract. MALT is composed of epithelial cells identified as follicle-associated epithelium or microfold cells (M cells) that act as the first mucosal barrier system and initiate the immune response [50].

**Figure 2:**
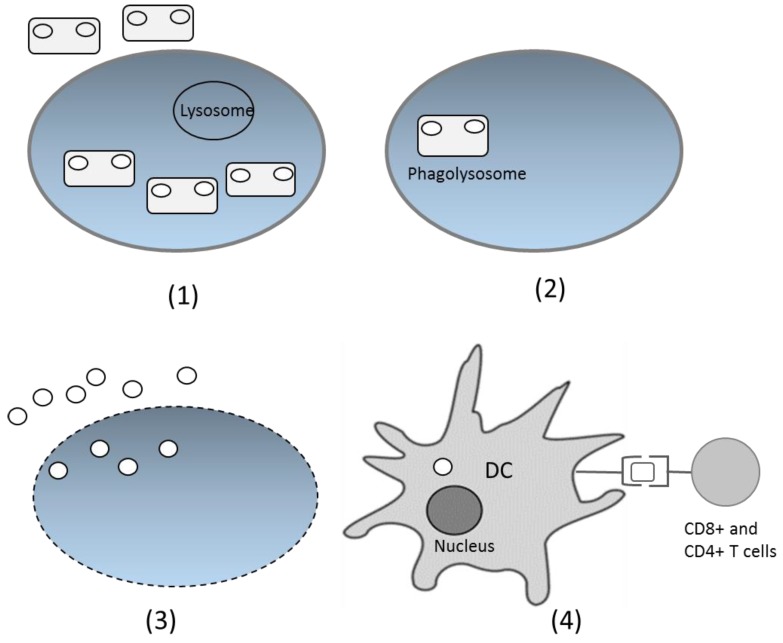
Proposed DNA vaccine delivery system using a live bacterial vector. (**1**) Bacteria are recognized by immune cells and phagocytized; (**2**) Inside the cells, bacteria fuse with the lysosome and form phagolysosomes; (**3**) Bacteria lyse and the DNA plasmids are released from cells; (**4**) DNA plasmids are engulfed by the dendritic cells (DCs), and inside the DCs, the antigen gene is expressed as protein, which will later be presented by class I or II MHC and delivered to CD4+ or CD8+ T cells.

After being delivered through the oral route, bacteria with the DNA vaccine enter the digestion system. On the intestinal surface, the bacteria are recognized by M cells in Peyer’s patches and spread to the lamina propia [48]. The bacteria have specific characteristics in the form of microbe-associated molecular patterns (MAMPs), recognized by particular receptors, such as Toll-like receptors and Nod-like receptors. This introduction induces the native immune response and increases the adaptive immune response. Phagocytized bacteria shape phagolysosomes and trigger cell lysis, which further releases plasmids inside the bacteria. Cell components, including plasmids, are released and engulfed by dendritic cells (DCs) [51]. Inside DCs, plasmids enter the nucleus through special compartments, and the antigen gene is expressed by the host expression system. The expressed antigen is presented by class I MHC and activates the CD8+ T cells. The antigen can also be expressed as extracellular protein, presented by class II MHC, and activates antibody production and the T helper CD4+ cell response [25,52] (Figure 2). However, the precise mechanism of DNA transfer by live bacterial vectors is not yet fully understood for many species. The suggested mechanism is based on the bacterial invasion properties [53]. Thus, some invasive bacteria, such as *Salmonella typhimurium* and *Listeria monocytogenes* are preferable carriers for DNA vaccines.

Briefly, as a DNA vaccine carrier, bacteria are divided into two major groups: non-pathogenic bacteria and attenuated pathogen bacteria. The attenuated bacteria that have been studied as the DNA vaccine carrier include *Salmonella* spp. [4,23,47], *Yersinia enterocolitica* [54], *Shigella* spp. [55,56], and *Listeria monocytogenes* [57]. Pathogen bacteria target the mucous membranes as their infection route and as a result, they are suitable for mucosal administration. However, the main disadvantage includes the likelihood of causing infection, particularly in infants and immunocompromised patients [58]. Therefore, non-pathogen bacteria such as lactic acid bacteria (LAB) [59,60] may be preferable for development as DNA vaccine carriers. A comparison of the properties of several strains that are commonly used as DNA vaccine carriers is presented in Table 1.

## 4. Lactic Acid Bacteria as the DNA Vaccine Carrier

LAB is an excellent candidate to be manipulated as a DNA vaccine carrier. LAB has been used in food fermentation for centuries and is a Generally Recognized as Safe (GRAS) organism [58,60]. LAB is also resistant to acidic conditions in the gastrointestinal (GI) system and is able to deliver the vaccine to the intestinal area [69,70]. Several LAB strains are famous as probiotic bacteria, such as *Lactobacillus casei, Lactobacillus delbrueckii, Lactobacillus acidophilus, Lactobacillus plantarum, Lactobacillus fermentum* and *Lactobacillus reuteri*. Probiotic bacteria reduce lactose intolerance symptoms, such as diarrhea and flatulence, which appear in lactose intolerant patients who consume milk. Probiotic bacteria also increase the immune response toward pathogens by inhibiting pathogen colonization in the GI tract [59] and promote the mucosal immune system by activating plasma cells, inducing secretion of immunoglobulin A (IgA) and migration of T cells [71].

*Lactococcus lactis* is the most-studied LAB since its genome is easily manipulated, and many genetic tools have been engineered for *L. lactis* [54,64]. Notably, one of the main advantages of using *L. lactis* as a DNA vaccine carrier is its ability to pass through the intestinal tract without colonization [60].

Some studies confirmed that *L. lactis* is able to transfer DNA plasmid to the host cells [24,72,73,74]. A study conducted using native *L. lactis* showed its ability to deliver DNA plasmid into mammalian cells. The coincubation led to the expression and secretion of transgene products [24]. Yagnik et al. showed that *L. lactis* is capable of transferring DNA plasmid to Caco-2 cells in the absence of chemical treatment or other invasive proteins [72]. Another study demonstrated that glycine treatment escalates DNA transfer from *L. lactis* to Caco-2 cells [74]. The ability of *L. lactis* to transfer plasmid to mammalian cells in vivo was confirmed by the delivery of plasmid by *L. lactis* to murine epithelial membrane cells, and the protein was effectively expressed by the mammalian cell expression system [73,75].

Several attempts have been made to increase the efficacy of *L. lactis* delivery to the inside of DNA host cells. pValac, a new plasmid, was successfully constructed and demonstrated its ability to be delivered to the interior of porcine kidney cell lines [76]. The major advantage of the pValac vector is its small size (3.7 kb) compared with the previously used plasmid pLIG (10 kB) [24,75]. A smaller plasmid allows the cloning of larger DNA fragments with an easier transformation process.

To improve internalization capacity, *L. lactis* that expressed *Listeria monocytogenes* invasin internalin A (InlA) or *Streptococcus pyogenes* Fibronection-binding protein A (FnBPA) was developed. Internalin A mediates internalization through a binding interaction with the E-cadherin expressed on human epithelial and endothelial cells. Analysis showed that both strains effectively delivered the DNA plasmid to Caco-2 cells and the target protein was expressed inside the Caco-2 cells [62]. Another study demonstrated that the *L. lactis* expressing internalin A was able to deliver DNA to dendritic cells, either directly or by passing through the epithelial mono layer [52]. Moreover, the combination of an invasive *L. lactis* strain expressed In1A and pPERDBY, a reporter plasmid with immunostimulatory properties, successfully increased the expression level of the target gene in Caco-2 cells. This study demonstrated that the new combination increased the target gene expression three-fold compared with the expression in the non-invasive *L. lactis* strain [63].

## 5. *Salmonella* spp. as the DNA Vaccine Carrier

*Salmonella* spp. is a Gram-negative bacterium that causes salmonellosis through orofecal routes. As a DNA vaccine carrier, *S. enterica* serovars Typhimurium (*S. typhimurium*) is the most widely used *Salmonella* spp. [4,64,65]. This bacterium is suitable for oral administration as its natural infection route. However, it can induce both mucosal and systemic immune responses, activating the humoral and cellular immune systems [66]. As pathogenic bacteria, *Salmonella* spp. induce the immune response through their lipopolysaccharides (LPS) and flagellin content on their surface that is recognized as pathogen-associated molecular patterns (PAMPs). Flagellin induces the immune response by binding with Toll-like receptor 5 (TLR5), whereas LPS binds to TLR4. The binding activates nuclear factor-kappa B (NF-kB) and the mitogen-activated protein kinase (MAPK) pathway, which is followed by the release of cytokines [64,65]. *S. enterica* virulence genes are encoded in *Salmonella* pathogenicity islands (SPI). The two main SPIs are SPI1 and SP2, which participate in host cell invasion and intracellular host cell survival, respectively [64]. Due to their remarkable pathogenicity, mutant *Salmonella* have been developed as DNA vaccine carriers.

Mutant *S*. *typhi* and *S*. *typhimurium* that were developed as vaccine carriers have an *aroA*, *aroC*, or *aroD* mutation. Through these mutations, *Salmonella* is not able to produce aromatic substances; therefore, it cannot replicate inside the host. However, the bacteria still invade the host intestines and survive long enough to induce an immune system response. Other mutant types have also been established, such as mutants that cannot produce guanine and adenine bases, causing distress in cell wall production [77], the DNA repair system, or virulence gene regulation [26,51]. Kong et al. successfully constructed a recombinant attenuated *Salmonella* mutant strain that has a hyper invasive phenotype that can invade the host cell, escape the endosomes, and reduce the bacteria apoptosis. As a consequence, the DNA is allowed to efficiently enter the nucleus [77]. Several other manipulations have been studied to improve the ability of *Salmonella* spp. as a vaccine carrier, including the manipulation of lipid A, outer membrane vesicles and engineering the dual-plasmid system, as described briefly by Wang et al. [67].

The *S. typhimurium* mutant was demonstrated to be a remarkable candidate as an anti-atherosclerosis DNA vaccine carrier. *S. typhimurium* (*aroA-*, *dam-*) oral administration that contained a plasmid with the *Flk-1* gene inhibited atherosclerosis and decreased aortic lesion size in atherosclerosis model mice. *Flk-1* is a vascular epithelial growth factor receptor 2 (VEGRF2)-encoding gene in mice. The vaccination activated T cells and inhibited neoangiogenesis, which is involved in atherosclerosis. The cellular immune response was detected through decreasing the expression of VEGFR2 in endothelial cells [23,78]. This approach was also used in another study conducted by Hauer et al. by manipulating *S. typimurium* with a plasmid encoding for TIE2, which is an angiopoietin receptor in the endothelial surface that contributes to the development of atherosclerosis. Oral vaccination in atherosclerosis model mice induced a cellular immune response, decreased atherosclerosis lesion, and stabilized plaque. The cellular immune response was measured by the decrease in the number of endothelial cells that expressed TIE2 in vaccinated mice [79].

A similar study, using a DNA vaccine encoding for CD99, was also successfully conducted. CD99 is a protein expressed in leukocytes and endothelial cells involved in leukocyte recruitment in atherosclerosis lesion areas. The CD99 vaccine was consumed orally with *S. typhimurium aroA-* as the carrier. The vaccine generated CD8+ T cells that lysed CD99 expressing cells, so fewer leukocytes were detected in the lesion area. The vaccination also decreased lesion formation by 69% in carotid arterial [80].

Liang et al. confirmed Δ*asd*/Δ*crp* mutant *S. enterica* successfully delivered the somatostatin gene in a mice model. The mutant lacks the antibiotic resistance gene and is based on the aspartate-semialdehyde dehydrogenase gene as the selection marker. Administered bacteria that contained a DNA vaccine effectively induced mucosal and systemic immune responses. The safety of the vaccine was shown by negligible integration of the plasmid gene into the host cellular genome [4].

## 6. Other Bacterial Live Vectors as DNA Vaccine Carriers

Other bacteria that were developed as DNA vaccine carriers include *Listeria monocytogenes* [65,81], *Shigella* spp. [51,82], and *Yersinia enterolica* [54].

*L. monocytogenes* is a Gram-positive bacterium that invades several cell types, such as mucosal epithelial cells, hepatocytes, macrophages, DCs and epithelial cells in the blood-brain barrier [25]. The bacteria invade and divide inside mammalian cells and consequently induce a high immune response. *L. monocytogenes* spread to other cells making it an effective DNA vaccine carrier against cancer. Given its ability to infect intestinal epithelium, *L. monocytogenes* has become an attractive candidate for oral vaccine delivery [57]. The disadvantage of this organism is the pathogenicity that leads to cholecystitis in human. Mice infected with *L. monocytogenes* showed bacterial colonization in the gall bladder. The mutant variant that was developed has mutations in the gene encoding for biotin metabolism (*lmo*0598) and ligase lipoate putative protein (*lmo*2566). The mutant variant was confirmed to induce an immune response, but did not replicate in the gall bladder [83]. Another study using mutant *rsΔ2 L. monocytogenes* showed that the bacterium, ingested orally, delivered a DNA vaccine containing the ovalbumin encoding gene. Increasing the antibody titer in a vaccinated mice serum demonstrated that the expressed antigen induced an immune response [57]. A different study indicated that attenuated recombinant *L. monocytogenes*, with a plasmid encoding for *Mycobacterium tuberculosis* antigen, was capable of inducing a robust cellular immune response in mice [84]. Conversely, a study conducted by Loeffler et al. revealed that *L. monocytogenes* induced a better immune response, when delivering recombinant antigen protein, than plasmid DNA. Antigens expressed by *L. monocytogenes* significantly increased the CD8+ T cell response, whereas the DNA vaccine carried by *L. monocytogenes* failed to induce a specific T cell response [85].

*Shigella* spp. are used as DNA vaccine carriers due to their ability to be retained in cytoplasm and evade endosomes, thus effectively delivering the DNA to the nucleus. *Shigella* also has a natural ability to target lymphoid tissues, triggering optimal mucosal and systemic immune systems. A study showed the efficacy of a DNA vaccine carried by mutant *S. flexenery* to attenuate human immunodeficiency virus (HIV) infection in a murine model. A single dose of the *S. flexenery* vaccine given intranasally induced a robust CD8+ T cell response [55]. A similar in vivo study demonstrated that a *S. flexenery* mutant successfully delivered the DNA vaccine encoding for the HIV gag gene. The DNA vaccine was intranasally delivered by recombinant bacteria inducing a cellular immune response comparable to a naked DNA vaccine given intramuscularly [56].

*Y. enterocolitica* is chosen as a DNA vaccine carrier because it can survive in host tissues for several days. During this period, the DNA vaccine is replicated in accordance with *Y. enterocolitica* proliferation, increasing the amount of DNA vaccine [54]. In a previous study, *Y. enterocolitica* was proven to deliver a DNA vaccine encoding for Brucella antigens bacterioferritin (BFR) and P39. The DNA vaccine induced antigen-specific antibodies and a Th1 response. The vaccinated mice showed resistance against Brucella infection [54].

## 7. Conclusions

DNA vaccines are facing challenges in terms of an effective delivery system that effectively targets the immune system. As live vectors, bacteria are new promising agents for vaccine delivery. Bacteria have unique natural characteristics that promote immune responses. However, the main concern about bacteria as vaccine carrier is patient safety. Although some attenuated recombinant strains have been developed, non-pathogenic bacteria such as LAB are considered more suitable DNA vaccine carriers.

## Figures and Tables

**Table 1:** Characteristics of live bacteria used as DNA vaccine carriers.

Bacteria	Advantages	Limitations	Strategies	Ref.
*Lactococcus lactis*	Non-pathogenic bacteria Non-colonizing bacteria Easy to manipulate	Since it is not a pathogenic bacterium, the ability to deliver the DNA is limited Unable to induce the cellular immune response	Manipulate the bacteria to express invasin protein (InlA, FnBPA) Combination of invasin expressed strain and immunostimulatory plasmid	[50,60,61,62,63]
*Salmonella* spp.	Able to induce both cellular and humoral immune responses Genetic manipulation is established	Possibility of reversion to pathogenic wild type	Development of several types of attenuated strains	[64,65,66,67]
*Listeria monocytogenes*	Able to invade several different cell types; therefore, can effectively deliver DNA Induces both cellular and humoral immune responses	Highly pathogenic, especially to immunocompromised patients	Development of several types of attenuated strains	[25,68]
*Shigella* spp.	Effectively introduces DNA to nucleus	Restricted host specificity inhibits the in vivo efficacy assay		[55,56]
